# Schools’ and teachers’ roles and challenges in supporting the mental wellbeing of refugee youths: a qualitative study with Swedish teachers

**DOI:** 10.1080/17482631.2021.2007568

**Published:** 2021-12-01

**Authors:** Serena McDiarmid, Natalie Durbeej, Anna Sarkadi, Fatumo Osman

**Affiliations:** a Department of Psychology, University of Waterloo, Waterloo, Canada; b Department of Public Health and Caring Sciences, Uppsala University, Uppsala, Sweden; c Department of Health and Welfare, Dalarna University, Falun, Sweden

**Keywords:** Mental wellbeing, refugee youths, teachers, schools, thematic analysis

## Abstract

**Purpose:**

Resettled refugee youths are increasingly entering host-country school systems and are at risk of poor mental wellbeing. Schools and teachers are often expected to provide psychosocial support to youths with refugee backgrounds, but the teachers’ views on this expectation are poorly understood. We investigated the question: What do Swedish teachers believe is the role of (1) schools and (2) teachers in supporting refugee youths’ mental wellbeing?

**Method:**

Four semi-structured focus groups were conducted with 30 Swedish educators from five schools. Interview transcripts were analysed and themes reflecting the key concepts were constructed using reflexive thematic analysis.

**Results:**

Seven themes were constructed. Three addressed the role of schools in supporting refugee youths’ mental wellbeing: Promoting Belonging, Offering Refuge, and Instilling Civic Literacy. Four addressed the role of teachers: Building Relationships with Students, Maintaining a Non-therapeutic Relationship, Connecting to Professionals, and Instructing in the Classroom.

**Conclusion:**

Teachers believe that both schools and teachers play an important role in supporting refugee youths’ mental wellbeing and each contributes in unique ways. However, schools and teachers are not always successful in supporting refugee youth and teachers reported facing challenges such as unclear roles and a lack of resources.

## Introduction

The number of people classified as refugees by the United Nations High Commissioner for Refugees has skyrocketed globally. Currently, an estimated 79.5 million people have been forcibly displaced and at least 40% are thought to be children and youth (United Nations High Commissioner for Refugees (UNHCR), 2020).

Sweden is a major destination for refugees, in part because asylum seekers have access to social services, like medical care and education, immediately upon arrival (Nyman & Vargar, 2020). Educational entitlements for asylum-seekers include access to free, high-quality schooling, a diagnostic assessment to determine educational needs within two months of enrolling, access to a “preparatory class” for up to two years, extra support learning Swedish, and free school lunches, among others (National Agency for Education, 2021).

Granting of asylum claims in Sweden peaked in 2016 with 67 258 permits granted, about 35% of which went to minors (Migrationsverket, 2017). In 2018, youth with a refugee background made up an estimated 5% of all students aged 13–15 and an estimated 8% of all students aged 16–18 (Denkelaar, 2018).

Refugee youths entering school systems in high-income host countries often have unique, short-term educational needs such as limited language abilities in the host language (Şeker & Sirkeci, 2015), limited literacy or interruptions to their prior schooling (Boyson & Short, 2012; UNICEF, 2016), and limited knowledge of the host country’s culture (Borsch et al., 2019). And critically, refugee youths often suffer from poor mental wellbeing. The prevalence of depression, anxiety, and post-traumatic stress disorder have all been found to be higher among refugee youths than youths in the general population (Bronstein & Montgomery, 2011; Fazel et al., 2005; Kandemir et al., 2018; Khamis, 2019; Kien et al., 2019; Salari et al., 2017; Scherer et al., 2020) and the same pattern has been observed among refugee adults (Lindert et al., 2009; Steel et al., 2009; Turrini et al., 2017). The cause of these increased rates of mental health problems may be due to the prolonged persecution and forced displacement that resettled refugees have, by definition, experienced. These experiences are often steeped in uncertainty, struggle, and violence, and exposure to such potentially traumatic events has been linked to mental health challenges (Rizkalla et al., 2020; Rosshandler et al., 2016; Steel et al., 2009). Given the large proportion of refugee youth in Sweden and the high prevalence of mental health challenges in this population, schools and teachers are undoubtedly interacting with and teaching refugee students experiencing poor mental wellbeing. And, according to the Education Act (Skollag) (2010), Swedish schools and teachers are obliged to support all students and provide equal access to and quality of education for all.

Several interventions targeting improved mental wellbeing among refugee youths have taken place in schools (e.g., Durbeej et al., 2021; Fazel et al., 2009) and these interventions have demonstrated that improved mental health outcomes can be achieved in schools (Tyrer et al., 2014). Schools are considered an ideal location for addressing mental wellbeing because they are a non-stigmatized location that are frequented by a majority of youths (Fazel et al., 2016). School is also the workplace of teachers who know refugee youths deeply and across a variety of contexts and who can therefore effectively support refugee youths (Tyrer et al., 2014). In fact, the roles and responsibilities ascribed to teachers are becoming increasingly complex around the world; they are stretching beyond instruction to encompass activities like psychosocial support and the fostering of social cohesion and belonging (Ministry of Education & Research, 2016; Schleicher, 2018).

## Understanding the role of schools and teachers through ecological systems theory

Bronfenbrenner’s (1977) ecological systems theory provides a useful framework for understanding the intersecting experiences and social environments that shape one’s development—including the mental wellbeing of refugee youths. In particular, this model highlights the interconnected nature of the systems in which a refugee youth lives. The ecological systems theory describes an individual as being influenced by—and having an influence on—all levels of the system in which they live. Teachers and schools are both considered to be parts of the youths’ *microsystem*: they are immediate relationships or organizations with which the student interacts, and youths’ development is thus influenced by both youth-teacher and youth-school interactions. For example, a refugee youths’ mental wellbeing may be influenced by the attitudes or practices of the teacher, and by the atmosphere of the school. Similarly, the behaviours of schools and teachers are shaped by the policies of the agencies governing education in the youths’ *exosystem*, and the larger cultural context or *macrosystem* in which the schools and teachers operate.

Bronfenbrenner (1977) detailed levels in which systems outside the individual interact to affect the individual. One is the *mesosystem* which describes the interaction between microsystems. For refugee youths, this includes interactions between the teacher and school. The teacher’s actions or beliefs may shape the school environment or functioning, while the policies or climate of the school may shape the teacher’s professional decision making. These changes will, in turn, influence the refugee youths’ development. Another level is the *exosystem* which describes systems that the individual does not participate in but that indirectly affect them through one or more microsystems. For example, the youth does not participate in the government but is influenced through their school microsystem by the governmental policies and laws that regulate schools and teachers’ responsibilities.

Pastoor (2017) has argued that the needs of refugee youths are complex and can’t be fulfilled by only one group, so research investigating several groups and levels, such as teachers *and* schools, is therefore needed. Using the ecological systems framework, we make connections between the actions of the schools and teachers in the *microsystem*, note the ways schools and teachers influence one another in the *mesosystem*, and tie in the influence of governmental policies through the *exosystem* and cultural beliefs through the *macrosystem*.

## Schools and teachers affect the wellbeing of youths with refugee backgrounds

Refugee youths’ wellbeing is influenced by their school environment. Previous work has shown that inclusive school environments—that is, environments that are psychologically safe, welcoming, non-discriminatory, and promote a sense of pride and belonging among its members—can support refugee youths’ mental wellbeing across social and geographic contexts in many high-income host countries. For example, according to refugee youths, school environments that are inclusive and non-discriminatory and that foster strong home-school ties support their achievement (Hek, 2005). Similarly, a longitudinal study found an inclusive school environment predicted higher social competence and academic achievement among young immigrants (Oxman-Martinez & Choi, 2014). A systematic review, including data from 1441 youths, also found that forcibly-displaced minors’ self-reports of a positive school experience—one in which the school felt safe and they felt they belonged—was associated with better mental health outcomes (Fazel et al., 2012).

Conversely, negative school environments can harm youths’ mental wellbeing. Many refugee youths have reported bullying at school (Correa-Velez et al., 2010; Guo et al., 2019; Hek, 2005) and bullying is associated with less happiness in refugee youths (Correa-Velez et al., 2010) and poor mental health outcomes for all youths (Moore et al., 2017).

The wellbeing of refugee youths is also affected by their teachers. High levels of support from teachers were associated with better mental health among newcomer youth (Cristini et al., 2011) and positive teacher-student relationships promoted social connections among a large sample of ethnically diverse youths (Thijs & Verkuyten, 2008). Intervention work has also demonstrated that improving inclusion efforts among teachers of refugee students is associated with better learning outcomes for those students (Bačáková & Closs, 2013). In qualitative work, refugee youths have reported that the presence of knowledgeable, specialist teachers and supportive teacher attitudes helped them to settle and achieve at school (Hek, 2005) and teachers who go to great lengths to help students reportedly foster students’ self-confidence and motivation to achieve (Thommessen & Todd, 2018).

Conversely, negative interactions with teachers are associated with negative outcomes for youth. Refugee youth in many studies internationally have perceived racism and prejudiced attitudes from teachers (Guo et al., 2019; Osman et al., 2020; Thommessen & Todd, 2018) and discriminatory teacher attitudes are associated with poorer student self-esteem, social competence, and academic achievement among immigrant youth (Oxman-Martinez & Choi, 2014), and poorer mental health outcomes (Montgomery & Foldspang, 2008; Fazel et al., 2012; Luthar et al., 2015). However, it should be noted that each refugee youth has a unique set of experiences and, while discrimination is immoral and has negative impacts for many, some individuals have reported feeling they had the power to challenge discriminatory attitudes and that these attitudes made them more motivated to succeed and achieve their goals (Thommessen & Todd, 2018).

Despite evidence demonstrating that schools and teachers affect the mental wellbeing of refugee students, there is little evidence with regard to what teachers believe the role of schools and teachers should be. Data on teachers’ beliefs about school is limited. One study from Pastoor (2015) interviewed refugee youths and teachers to investigate whether school plays a role in the “psychosocial transitions” refugee youths make when settling into the host country. Pastoor found schools do play a role by providing opportunities for socialization, integration, and health and wellbeing promotion. Yet, more data is required for a robust understanding of a school’s role in supporting mental wellbeing. Evidence regarding teachers’ views on their own roles is similarly lacking and consists mainly of incidental data generated from teacher interviews about the needs of refugee students. For example, in interviews reported by Şeker and Sirkeci (2015), teachers reported refugee children often experienced interpersonal conflict with native schoolmates and, consequently, described feeling responsible for promoting social cohesion within classes. But questions still remain about the role of teachers and how their role may intersect with the role of schools.

It is clear that schools and teachers in high income host countries are becoming key players in supporting refugee youths’ mental wellbeing, often even supporting interventions meant to improve mental wellbeing among refugee youths. Yet there is a gap in the literature when it comes to understanding what *teachers* believe about the role of schools and teachers in supporting refugee youths’ mental wellbeing. If schools are to continue to be a common location for mental health interventions, and if teachers are expected to be involved with the support of refugee youths’ mental wellbeing, it is necessary to address this gap. This study does so by exploring the question, “What do Swedish teachers believe the role of (1) schools and (2) teachers to be in supporting the mental wellbeing of refugee youths?”.

Addressing this question and gap in the literature will have several benefits. First, it will inform professionals and researchers concerned with the mental wellbeing of refugee youth by providing novel information about the ecological systems in which these youth exist. Next, data will inform interventionists and mental health practitioners who aim to engage teachers as stakeholders in school-based interventions. And finally, data will inform educational policy makers who, in understanding the views of education workers, will be better able to ensure the effective functioning of the school system.

## Methods

### Design

This qualitative study had an exploratory design, based on a thematic analysis to provide knowledge about what Swedish teachers believe the role of (1) schools and (2) teachers to be in supporting the mental wellbeing of refugee youths. A qualitative exploratory approach is well-suited to situations in which little to no data currently exists and researchers use a primarily inductive approach to explore a broad research question (Rendle et al., 2019).

Data from four focus groups with 30 Swedish teachers were used. Focus groups were selected as the ideal method for data collection because they facilitate investigations of a phenomenon from many different viewpoints (Krueger, 2014). Furthermore, focus groups allow for an understanding of how teachers *collectively* view their role and the role of schools in supporting refugee students’ wellbeing. However, focus groups have the disadvantage of being dependent on the skills of the moderator and susceptible to domination by a few especially vocal participants (Green & Thorogood, 2014).

### Setting and participants

A strategic sample of teacher participants were recruited for focus group discussions from target high schools (7th—9th grades and introductory classes) in which a larger, ongoing study was taking place (Durbeej et al., 2021; funded through the European Union’s Horizon 2020 research and innovation programme under grant agreement No 754849). Teachers were identified as an ideal population with whom to conduct focus groups because they work on the frontlines of schools and can best articulate the role they feel they play. Teachers are also part of the educational institution and can therefore better illuminate the role of the schools.

Target schools were located in a mix of rural, suburban, and urban areas but all were considered multiethnic as ≥ 30% of the school population were newcomers. Information sessions about the larger project were held for teachers at their school of employment. Sixty-three educators from five schools attended an information session, often at the direction of school administration. Of those, 45 were still teaching at the school when the research took place and chose to participate in the focus groups.

Focus group participants were all employed by a target high school in a role in which they supported student learning. In describing participants, we use the terms “teacher” and “educator”, though the nature of their roles in schools varied. Some held formal teaching certifications and worked as instructors while others did not have the formal certification and worked one-on-one or in smaller groups with students in a mentor or translator-like role. Educators also had varied personal backgrounds: some were native to Sweden while others had immigrated to Sweden or arrived as a refugee themselves.

### Data collection

Focus groups discussions took place in October 2019 on the day teachers were participating in a larger training session. Teachers from one school participated on one day (*n* = 3). Teachers from the remaining four schools took part simultaneously on a second date. While 42 teachers agreed to participate on this date, only three trained moderators were available to facilitate focus groups. Therefore, five groups were formed and three were randomly selected to participate in the study by taking part in moderated focus groups (*n* = 27) while two groups did not participate in the study and instead took part in an unmoderated discussion with their colleagues (*n* = 15). Ultimately, the total number of participants in this study was 30.

Discussions lasted one hour and were conducted by a moderator. An observer was also present during focus groups 1, 2, and 4 (see Table I). The observer took notes about the qualities of interactions between participants including any perceived power imbalances and participants’ level of engagement. In all, three unique moderators and three unique observers were involved.

**Table I.** Focus group characteristics

Focus Group	Participants	Schools Involved
1	9 participants (7 women, 2 men) plus one moderator and one observer	Four schools from a mid-sized urban municipality
2	9 participants (5 women, 4 men) plus one moderator and one observer	
3	9 participants (4 women, 5 men) plus one moderator	
4	3 participants (1 woman, 2 men) plus one moderator and one observer	One school from a mid-sized rural-suburban municipality

Focus groups 1, 2 and 3 took place simultaneously and consisted of 9 participants each. Participants included teachers from 4 different schools all in a mid-sized urban centre with a large immigrant population. Teachers from these different schools did not know each other prior to the discussions. Focus group 4 took place one day prior at a mid-sized school with a catchment area that was both rural and suburban. This focus group included 3 participants who were all educators at the same school and known to each other.

Data were collected using a semi-structured, predefined interview guide. Questions related to two key topics: wellbeing within schools, and wellbeing within a broader social context (see supplementary information for questions). Two questions focussed on teachers’ expectations of their forthcoming training and responses to these questions were also analysed as they still reflected teachers’ beliefs related to our research question. The order of questions was generally kept the same between groups but the moderator sometimes omitted questions or skipped ahead based on time constraints. Follow-up probes were used as needed to clarify participants’ responses or follow a line of inquiry that arose during discussion. No school administrators were present on the day of the focus groups and participants were encouraged to maintain privacy regarding the material discussed. Group dynamics were highly positive and educators appeared excited to participate. They talked extensively and, in many cases, discussions were so extensive that not all interview questions could be asked, but both topics in the interview guide were always addressed. We noted a convivial feeling among participants who would joke with each other or ask each other for input. However, participants did not always agree and seemed comfortable expressing different or dissenting ideas. Cases of disagreement are discussed in the results section. We did not observe a difference in the responses provided by participants of different genders, backgrounds, or roles within the school based on observer field notes.

All discussions took place in Swedish. While we know many educators spoke Swedish as an additional language and that discussion language can impact the quality of data among minority participants (Sills & Desai, 1996, July), all educators were employed in jobs that required fluent Swedish abilities. Thus, their Swedish was deemed sufficient for full participation in the discussion.

### Data analysis

Audio-recorded focus group discussions were transcribed verbatim and translated by a research assistant into English. Transcripts and translations were checked against the original audio-recording by author FO.

English transcripts were analysed using reflexive thematic analysis (Braun & Clarke, 2006, 2019; Clarke & Braun, 2013). Analysis began with transcripts being read inductively many times. Data were then read critically to derive codes that captured the key ideas and concepts expressed by participants. The codes explicitly focused on the core concepts identified in our research questions: what schools and teachers are doing to support newcomer students and the challenges they face in providing this support. Codes and associated text segments were then organized further and grouped to construct themes in a reflexive manner. Themes reflect “patterns of shared meaning” with a central, uniting core concept (Braun & Clarke, 2019). As recommended by Nowell et al. (2017), credibility was established by first and last authors verifying the theme construction and by debriefing with our broader research team to provide an external check on the research process.

Thematic analysis takes an interpretivist stance and is a flexible method useful for understanding the concerns and perspectives of a group (Braun & Clarke, 2006). It is a well-documented approach in the social sciences (Nowell et al., 2017). Braun and Clarke (2019) note that researchers draw on their relevant expertise to construct meaningful themes: SM is a school teacher who has worked with refugee youth in education and community contexts in Canada while FO is a nurse who has worked extensively with refugee youths in educational, community, medical and research contexts in Sweden and arrived in Sweden as a refugee. While the process of two authors discussing and agreeing on the themes increases the overall credibility of the results, the fact that both authors have very different backgrounds also contributes to increasing the validity and robustness of the results.

### Ethical considerations

The study received ethical approval from the Regional Ethical Review Board at Uppsala University (dnr: 2019–031160). All participants were informed about the nature of the study through oral descriptions and a written informed consent document and all gave their consent to participate. Participants were advised orally and in writing that their participation was voluntary and that they could withdraw their consent to participate at any time without penalty or explanation. Participants were also informed that their data would be treated confidentially by researchers but that confidentiality around focus group discussions could not be guaranteed due to the potential for other participants to break confidentiality.

## Results

Seven themes were identified from the thematic analysis: Promoting Belonging, Offering Refuge, Instilling Civic Literacy, Building Relationships with Students, Maintaining a Non-therapeutic Relationship, Connecting to Professionals, and Instructing in the Classroom. The first three themes describe the role of schools in supporting newcomer’s mental wellbeing while the latter four themes describe the role of educators. While the themes are connected, they represent distinct roles played by schools and teachers.

### The role of school in youths’ wellbeing

Swedish educators were asked whether schools play a role in supporting students’ mental wellbeing and their answer was unequivocally, “Yes.”

**Female Respondent (FR):** “The school plays a big role in helping students. Newly arrived or other … Swedish students as well. [It’s] a second home.” (Focus group data (FGD) 2)

Students spend the majority of their day at school—it is a “second home”—so the environment of the school inevitably shapes their mental wellness. We analysed the participants’ discussions and identified three themes which describe the role of schools in supporting newcomers’ mental wellbeing: Promoting Belonging, Offering Refuge, and Instilling Civic Literacy.

#### Promoting belonging

According to the Swedish educators, schools are not just buildings. They are communities formed by the collection of students and staff members who make up the school population. The community formed by a school is just as important to the mental wellbeing of its members as the physical space the school provides. In fact, teachers overwhelmingly described schools as providing students with a sense of belonging and community.

Schools support the forging of these social bonds by creating opportunities for interaction among students. School social clubs, sports clubs, and even homework help groups are prime opportunities for youth to meet and bond with others. Teachers also noted how amenities within schools, such as couches and ping pong tables, create physical spaces in which students come together and connect.

**FR:** “ … Sometimes they [students] do not want to go home during the day. We almost have leisure centers. There is ping-pong … They do not want to be off school. We have a summer school and it is full of students there. They like to stay.” (FGD3)

Teachers frequently described the community within the school as equitable and providing a feeling of “safety”. They emphasized that newcomer students are not made to feel like “others” which contributes to their sense of belonging.

**FR:** “ … here everyone is equally valuable, and everyone has the same rights to be here on the same terms …. here everyone has a place to be and here everyone feels safe, regardless of who you are and what background you have. It’s a pretty amazing environment.” (FGD1)

However, some teachers pointed out that, while schools *should* be a place where students feel they belong, this ideal is not always met. Some refugee students fail to form strong relationships with others, especially native students. Teachers described several possible reasons including cultural or language barriers, a low desire of native students to invest in relationships that may be ended if the newcomer is relocated by the Migration Authority, or newcomers’ anti-social behaviours stemming from mental illness.

#### Offering refuge

In the focus groups, participants spoke with great compassion about the pressures and responsibilities facing newcomer students. They frequently cited high family expectations, providing childcare to siblings, mental health issues within the family, issues with the Migration Authority, and uncertainty over their future as major causes of stress for youth. However, school was described by teachers as a place where students experienced a refuge from these stresses and responsibilities.

**FR:** “. … school can be a place where you can be yourself and recover and take a break from everything.” (FGD2)

Within the refuge of school, students were described as being able to focus on themselves and their individual growth—their personal wellbeing, interests, and desires—and ultimately improve their wellbeing.

**Male Respondent (MR):** “ … What I want to do is help them [students] find themselves. School is not everything. I want them to get to know themselves and know what they really want and not just lots of grades, points and high school. But rather know ‘who I am’ … ” (FGD3)

This idea of school as a refuge was contrasted with teachers’ views of the students’ home environments.

**MR:** “ … Many of the newcomers had difficulty studying at home—wrong conditions. Maybe a small apartment, two little siblings, parents who are not feeling well. They couldn’t work undisturbed.” (FGD2)

Teachers believed that home is not a place of peace for most students and is instead where students experience much of the stress that negatively affects their mental wellbeing.

#### Instilling civic literacy

Alongside its role in promoting belonging and offering refuge to refugee students, school was also described by teachers as an environment in which newcomers develop the knowledge, skills and attitudes to be productive members of Swedish society, thereby becoming “literate” in Swedish civics.

**FR:** “ … It is stated in the curriculum that you should bring light to the student’s health in different ways and make them grow as individuals to become future citizens. So it’s clear that schools play a huge role.” (FGD4)

Educators felt that instilling civic literacy was necessary to support the mental wellbeing of refugee students because, on arrival in Sweden, these students were missing cultural knowledge needed to become productive citizens. Teachers described this missing knowledge as causing newcomer students distress when they didn’t understand the “hidden culture” (FGD3) of society or when they experienced “culture clashes” (FGD2) because of misunderstandings about the Swedish way of life. Teachers noted that newcomer students commonly misunderstood democracy to mean freedom to do anything without consequences. Several teachers described students becoming frustrated (or causing frustration for others) when their understanding of democracy did not align with the understanding of democracy in larger society.

**MR:** “I think many refugee children misinterpret democracy. They think that democracy is freedom, so they don’t take responsibility for their actions. They think they can do anything. But they don’t take responsibility for it.” (FGD2)

Teachers described students as being instilled with civic literacy through implicit learning that occurs during students’ immersion in the supportive school environment and through repeated observations of how things are done in the Swedish context. Teachers felt this process of instilling civic literacy was necessary for the long-term wellbeing of students so they could have enough knowledge about Sweden’s civics and society to be able to make good choices for their future. However, teachers described how becoming civically literate can be a challenge for newcomers students who must figure out how to “feel, think and do” (FGD2) using the input they get at school which may be at odds with the input from their family or friends.

### The role of teachers in youths’ wellbeing

During the focus groups, educators discussed at length what they and their colleagues do to support the wellbeing of refugee youths in their schools. They described their roles, their interactions with students, and the challenges they and their students face. They felt their role in supporting refugee youths’ mental wellbeing was large and meaningful. One teacher stated, “We raise, we educate, and we develop.”. In analysing teachers’ discussions, we identified four themes that describe how educators support refugee youths’ mental wellbeing: Building Relationships with Students, Maintaining a Non-therapeutic Relationship, Connecting to Professionals, and Instructing in the Classroom.

#### Building relationships with students

Overwhelmingly, teachers reported that building relationships with students was an important role they played in supporting the wellbeing of refugee youths. Teachers described how they are often the most consistently present adult in the youth’s life and frequently take on a parental role. By demonstrating a genuine interest and care for the students they build trust and are often the person youth turn to for advice or to share their feelings. Teachers reported trying to raise the self-esteem of students too by providing encouragement and directing refugee students not to compare themselves to others, especially native students who were ahead academically. Educators who spoke students’ first languages tended to be especially sought out by students, according to teachers.

**FR:** “ … it’s about building a relationship with mutual respect where you may find some common interests. Now I’m not extremely interested in football, but I’m usually into it with some students. … I strongly believe in creating a social relationship with the student and to be able to help the student—‘Look here what you can do’—and build up the student … I think back to when we had that big stream of refugees. They were very happy when we hugged. Not everyone wanted to, but most thought that a hug could mean a lot. I could get into a maternal role … ” (FGD4)

Based on the deep bonds formed between teachers and students, teachers reported hoping students would feel comfortable sharing their personal histories, especially if those stories were traumatic. Teachers conveyed their belief that sharing these stories was an important part of students’ development and would ultimately improve their mental wellbeing. Teachers described how they intentionally created opportunities in which students could comfortably share but never pressured the students to do so.

**MR:** “Sometimes you have to find a key to the student’s heart so that the student opens up to us. It is very important that the student does not close everything in their heart … ” (FGD 4)

However, students’ disclosures often left teachers feeling apprehensive. Many teachers expressed concern over whether they were the proper person to be offering advice and emotional solace to students and worried that their responses might do more harm than good (see “Maintain a Non-therapeutic Relationship”). They also expressed concerns over being able to meet the needs of so many students simultaneously, and worried about students “falling through the cracks.”

**FR:** “They need more support and it is difficult to have time to give them that support. Then it’s so awfully little time. A lesson goes so fast and there are so many students. It’s so damn hard to have time.” (FGD3)

Teachers further described how, through these relationships with students, they developed a deep knowledge of students’ personal histories. Teachers used this information to identify opportunities to connect students to outside organizations (see “Connecting to Professionals”) and also to inform and tailor their teaching practices to the student (see “Instructing in the Classroom”).

#### Maintaining a non-therapeutic role

While teachers strongly and unanimously agreed that forging strong, caring relationships with students was an essential part of their role, they also clarified that they are not mental health specialists, nor is school a place for mental health treatment.

**MR:** “We work with learning—then we can help. But we are not a therapy unit.” (FGD1)

Yet teachers frequently described scenarios in which they were put in the position of needing to offer therapy-like emotional supports to students. Reflecting on these situations, teachers said they feared they were not the right person to support students through complex mental health challenges. They were aware they did not have the training of mental health experts and worried their efforts could cause harm to the student.

**MR:** “I thought, where does the limit go to our responsibility when a student is not feeling well? Because it’s so easy to take on everything. It’s MY student who’s not feeling well. And we who work as teachers are solution-focused. That is, when someone comes to me and says ‘I can’t concentrate’ … I do not say ‘you have to learn to concentrate’ but I say ‘what is the reason?’. ‘I’m tired’, ‘Okay what’s the reason?’. ‘Because I’m not feeling well, I cannot sleep.’ ‘Why don’t you feel good?’ So it is easy that you kind of want to take it all yourself. And I think it’s very difficult to know who to turn to.” (FGD3)

However, teachers also feared that if they did not provide emotional support to struggling students then no one would, resulting in greater harm to the student. Many teachers discussed the lack of timely responses from other elements of students’ support systems (e.g., mental health care teams or a government-provided legal guardian) and described students as getting “stuck in the piles” of paperwork on other professionals’ desks. Teachers also noted the wide variability in the quality of services provided by professionals outside the school system, describing some as passionate and committed, while others “just want to raise money”. This suggests teachers may also fear the professionals to whom they refer their students will not show the same level of care for the student that the teacher would show.

#### Connecting to professionals

Drawing on their personal knowledge of students’ mental health needs (see “Building Relationships with Students”), teachers described connecting with other professionals to get additional information or outside support to improve students’ mental wellbeing.

**MR:** “We are the ones who know how to navigate Swedish society. They [students] often want help with that.” (FGD3)

Teachers reported connecting with staff in the school (e.g., tutors or school counsellors), professionals providing social services outside the school (e.g., government-appointed guardians), and staff in community organizations (e.g., staff at football programmes). In some cases, the professional provides the teacher with the information s/he needs to meet the mental health needs of the student, such as a fellow teacher sharing strategies that have worked for them when interacting with a student. In other cases, the professional provides services to the youth directly, such as in the case of a mental health professional or football coach.

**MR:** “There are many [students] who come to me and say that they want to start playing basketball or football. Now I am fairly new in [the city name] so I don’t really have those contacts. But then I advise to “Talk to this person, he is playing football”, or “He is a leader here.” (FGD3)

A notable instance of “connecting” described by teachers was the act of making a concern report to the local Child Services department. Teachers are bound by law to report suspicions that a student is experiencing harm (e.g., domestic abuse or child neglect). Some teachers described making these reports often. From their descriptions, we noted they seemed motivated by a sense of legal obligation, an attitude that unnecessary investigation caused little harm while the lack of a necessary investigation could have grave consequences, and a feeling that this was one of the few ways they could be assured a student’s needs would be addressed.

**FR:** “But if you are really worried—we have a civil servant responsibility—then you have to report a concern. So it is the case that you experience that someone is in danger, you know, they tell stories from home, for example. … And I mean … It’s not about us knowing for sure, it’s about the slightest suspicion, so you should report a concern. I make several concern reports every school year.” (FGD3)

However, there were other teachers that seemed shocked by the liberal use of reporting and expressed reluctance to make reports.

#### Instructing in the classroom

While teachers are building relationships with students, connecting them to professionals, and navigating the struggles of not providing therapy, they still spend significant time instructing students. Teachers described providing direct instruction on mental wellbeing. For example, some teachers explicitly instructed students on strategies for improving sleep. Several participants were physical education teachers and also viewed their instruction on physical fitness and sexual health as contributing directly to improved mental wellbeing. Teachers noted that spending time teaching students to achieve wellbeing seemed a necessary prerequisite to providing effective academic instruction.

**FR:** “ … if you do not feel well, then there will be no learning. It is the basis for us to be able to do the other.” (FGD1)

Even when teachers were not explicitly instructing students on wellbeing, their lessons still supported mental wellbeing by accommodating each student’s unique needs. Teachers noted that they provide accommodation to all students but newcomers typically require a wider range of accommodations because of the high variability in their prior life experiences.

**FR:** “We have everything from those who have gone 9 years in school to some who never went. It is an incredible range to satisfy. It’s difficult to meet everyone’s demands, but then you have to try to meet everyone somewhere in the middle.” (FGD 4)

Teachers most often mentioned accommodating students academically. For example, they might simplify material or teach more slowly, thereby reducing students’ academic stresses and supporting their wellbeing. But in rare cases, teachers also mentioned accommodating students’ cultural needs. For example, a teacher described accommodating Muslim students who do not dance by adapting her dance lessons into “collaborative training exercises” set to music. These efforts support the wellbeing of students by increasing their opportunities for participation and creating a more inclusive classroom environment.

## Discussion

This study provides a unique investigation into teachers’ beliefs about the role of schools and teachers in supporting refugee youths’ mental wellbeing. Taken together, the results indicate that teachers *do* believe schools and teachers play an important role in supporting refugee youths’ mental wellbeing, that schools and educators provide support in specific ways, and that there are challenges schools and teachers face in performing these roles and providing the level of support refugee students need. We also note how the different ecological systems within a refugee youths’ life interact to influence their mental wellbeing and the roles teachers and schools can and cannot play.

### The role of schools

In regards to the role of schools, we noted that teachers conceptualized “school” broadly and considered school to include the school’s physical buildings, the community of students and staff within the school, and the educational curriculum and policy held by the school institution.

Teachers in our study most strongly identified schools as promoting a sense of belonging among youths, and teachers described how feelings of belonging were supported through schools’ social environment and the physical spaces within the school. This is consistent with considerable prior evidence about the importance of a sense of belonging in schools on youths’ mental wellbeing (Fazel, 2015; Pastoor, 2017; Kia-Keating & Ellis, 2007; Osman et al., 2020; Şeker & Sirkeci, 2015; Thapa et al., 2013). Furthermore, previous work has similarly described how refugee youths’ engagement in social activities and informal socializing—opportunities that teachers in this study believe schools should provide—can facilitate social connections and foster belonging (Fazel, 2015; Pastoor, 2017). Teachers’ reports about how the physical space within the school acted to promote belonging is also consistent with recent work from Fejes and Dahlstedt (2020) that described how the physical placement of refugee youths’ learning spaces on school campuses contributes to their feelings of inclusion or exclusion.

Educators also described schools as providing a refuge from outside responsibilities, allowing refugee youth to focus on their personal betterment and development. To our knowledge, no similar finding has previously been identified. This belief may be unique to contexts with a *macrosystem* like Sweden that promotes individualistic cultural ideals. Individualism is widespread in Swedish culture, specifically in citizens’ private lives (Tian & Guang, 2017) and increasingly, within the teaching profession (Erlandson et al., 2020). Other cultural contexts, especially those that identify as collectivist, are unlikely to place the same emphasis on youths’ personal development. Therefore, teachers in other global contexts may hold different views on the role of schools, as might subgroups of teachers within Sweden, such as those in privately-run international or religious schools.

Finally, teachers described schools as playing a role instilling civic literacy and shaping refugee youths into civically-engaged and informed citizens. This belief likely stems from the cultural importance placed on democracy within the Swedish *macrosystem*, and from the governmental *exosystem* which, through the Swedish Education Act (Ministry of Education & Research, 2016), mandates that “ … schools shall convey knowledge and values.” (p. 75) and, “[student’s] education shall also convey and entrench respect for human rights and the fundamental democratic values upon which Swedish society rests.” (p. 114). The desire to correct “misunderstandings” of democracy among students may also be rooted in an ethnocentric view that students’ cultures are not sufficiently democratic, consistent with prior literature (e.g., Guo et al., 2019; Osman et al., 2020; Thommessen & Todd, 2018).

Pastoor (2015, p. 2017) has noted similar findings regarding the schools’ role in instilling civic literacy in a Norwegian context: she described schools as playing a role in “mediating” refugee youths’ wellbeing during resettlement, and instilling civic literacy may be one way in which schools mediate this relationship (Pastoor, 2015). Furthermore, Pastoor’s (2017) later work reported that refugee youth can go from peripheral to fully participating members of their communities through the process of enculturation and learning about the society’s social and civic practices. This learning can support success in Swedish society, which in turn leads to greater acceptance by others (Pastoor, 2017) and increased wellbeing.

Teachers reported that the primary challenge facing schools was in translating the *promotion* of belonging in schools into actual feelings of belonging among students. Newcomer and native students were often described by teachers as socially separating themselves, which teachers believed reflected a lack of belonging for newcomers. Teachers described several challenges that might contribute to newcomers not feeling belonging including language and cultural barriers, perceived discrimination or lack of interest from the native students, or a lack of opportunity for interaction which is consistent with prior work (Pastoor, 2017; Osman et al., 2020; Şeker & Sirkeci, 2015). A similar lack of belonging has been reported among other refugee youths and is often characterized by experiences of bullying or discrimination (Correa-Velez et al., 2010; Guo et al., 2019; Hek, 2005).

### The role of teachers

Teachers reported that they and their colleagues also play an important role in supporting the mental wellbeing of refugee youths’ and the ecological systems theory supports the idea that refugee youths would be affected by their interactions with teachers. In describing their role, teachers overwhelmingly identified building supportive relationships as the major activity through which they support refugee youths’ mental wellbeing, and they specified that these relationships should remain non-therapeutic because teachers are not therapists. Additionally, teachers described their role as connecting students to other professionals for services that would support their wellbeing and instructing students on content and/or in a manner that supports wellbeing.

Teachers’ focus on their relationship with students is consistent with other research that overwhelmingly describes the teacher-student relationship as affecting students’ wellbeing and achievement (Cristini et al., 2011; Hek, 2005; Hoot, 2011; Thommessen & Todd, 2018; Thijs & Verkuyten, 2008). Interestingly, teachers focus on providing an opportunity for youth to share their personal history may be an especially vital service. Thommessen and Todd (2018) interviewed participants who arrived as refugees in their youth and the participants felt they would have benefited from being given the opportunity to share their past experiences with teachers, especially because many felt they could not discuss these challenges with their parents.

Teachers in this study spoke primarily of the ways teacher-student relationships support youth. But other work, especially work that includes student participants, has described how negative teacher-student relationships can be damaging to refugee youths’ mental wellbeing, especially if the student feels the teacher holds prejudiced or discriminatory view towards them (Guo et al., 2019; Osman et al., 2020; Thommessen & Todd, 2018). Notably, teachers in our study *all* felt developing relationships was a necessary role. This is in contrast to Mock-Muñoz de Luna et al.’s (2020) interviews with Swedish teachers that found a subset avoided learning about their refugee students’ background because it was emotionally difficult and instead chose to construe this information as outside their professional responsibilities.

A further belief of teachers was that the strong relationships they feel responsible for developing with students should remain non-therapeutic. This is consistent with other reports from Swedish teachers that educators do not provide healthcare for mental health problems but that mental healthcare needs exist for refugee children in the classroom (Mock-Muñoz de Luna et al., 2020). However, in our own data we observed a contradiction: while teachers *said* they believe they should not be providing therapy-like support, they often described situations where they appeared to be doing just that. They did not remark on this contradiction and it was not clear if they felt there was a contradiction between their stated belief and the actions they described. We did hear teachers describe the boundaries of their role as being blurry. Many teachers also shared concerns that youth would not receive the timely emotional support from a caring adult unless provided by the teacher. These data suggest that, while teachers believe they should not be providing therapy, they may sometimes fill the role of social services or therapists due to inadequate access to services and/or an unclear understanding of the boundaries of their role. This finding is consistent with other research that finds teachers are increasingly expected to provide psychosocial support to students (Ministry of Education & Research, 2016; Schleicher, 2018) and calls have been made to reconceptualize the role of teachers given these emerging psychosocial support expectations (Pastoor, 2015).

In discussing their role, teachers reported that the roles they play to support newcomers are largely the same roles they play to support native students. Indeed, it could be said that all good teachers build strong relationships with students, connect them to needed services, and adapt their instruction to best suit student needs. This suggests that teachers’ beliefs about supporting *refugee* students’ wellbeing may be easily generalized to supporting the wellbeing of *all* students. However, teachers did describe fulfiling their roles in slightly different ways for newcomers. Specifically, some teachers stated that they take more care, have more compassion, or are more lenient with newcomers because they are more aware of the personal struggles refugee students have experienced. Refugee youths have previously reported that their teachers hold lower expectations for them, and the youth have often attributed this to teachers being discriminatory or holding a low opinion of the student (Guo et al., 2019; Osman et al., 2020). Our observation provides an interesting alternative perspective that low teacher expectations—while experienced by youth as harmful—are actually motivated by compassion.

Educators also discussed challenges they face in supporting young refugees’ mental wellbeing. The most common barriers reported were: uncertainty about their responsibilities for supporting youths’ emotional health, a lack of external support from social services, the variable quality of external social service supports for students, the overwhelming level of need among students, the high variability in the types of needs they encounter, and the emotionally taxing nature of supporting students’ mental wellbeing. Teachers also noted that they had received little to no refugee-specific pedagogical training. Teachers described these challenges as affecting their personal mental wellbeing which made it difficult for them to provide sustained support to students.

Finally, while we did not specifically ask teachers about refugee students’ parents, we found participants frequently raised high parental expectations as a factor contributing to poor mental wellbeing among refugee youths. Teachers described parents as expecting students to consistently receive above average grades and pursue traditionally prestigious career paths. Teachers expressed distress that youth were pressured to pursue educational paths that may not be well-suited to their strengths or interests, once again demonstrating the influence of *macrosystem* level cultural beliefs about individualism on many teachers. This finding was notable due to the frequency with which it was discussed and because it was revealing of the way teachers see their own role. Many teachers related anecdotes in which they met with the parents and youth. In these meetings, they often observed the parent pressuring the youth to excel academically and contradicting the youth’s desires for their future. While teachers indicated they disagreed with the parent’s actions, no teacher reported raising concerns with the parent or intervening on the youths’ behalf. This suggests that, while teachers do support students by building relationships, there are boundaries to where and when they provide this support. Teachers did not appear to feel navigating intra-family conflicts was part of their role.

### Strengths and limitations

The design of this study provided numerous benefits. The use of focus groups led to more natural interactions between participants in which teachers posed questions and explained themselves to each other (Morgan, 2002). This circumstance leads to richer data and allows researchers to understand how educators talk about and understand the research topic *among themselves*. Discussions that occurred during focus groups may also have led participants to recognize or frame as significant their ordinary, everyday experiences that they may not otherwise have thought important enough to share (Green & Thorogood, 2014). The choice to include teachers, especially teachers from multiple schools and settings around Sweden, was also a benefit as it meant data were based on a variety of first hand experiences from those working with refugee youths. The results are therefore expected to be applicable to educational settings broadly.

There are also limitations to the study design. First, by working with transcripts translated from Swedish into English, some nuance in participant ideas may be lost (Wong & Poon, 2010). This limitation was mitigated by having the last author review the translations to ensure the meaning of the data was intact following translation.

Second, the use of focus groups can also have drawbacks due to their interactive nature and use of groups (Green & Thorogood, 2014; Morgan, 2002). For example, participants may be reluctant to share dissenting ideas or sensitive material in the group context. School administrators were not present during discussion which helped to address privacy concerns. We also noted that teachers appeared at ease discussing a range of topics and that they sometimes disagreed, demonstrating comfort in sharing different opinions than those of the larger group. But we cannot know if there were other sensitive topics that teachers chose not to discuss with the group.

Additionally, data analysis was conducted by a teacher (SM) and a nurse (FO). While our knowledge and life experiences have allowed for a deeper understanding of the data and a more rich analysis, we note that we may have been less attuned to details within our data that other professionals like social workers or policy analysts may have found most relevant. However, we feel the analysis is generally robust as both researchers came to agreement over the constructed themes and came from two different professions, cultural backgrounds, and geographic locations. Themes were also verified by the other two authors: a behavioural scientist (ND) and a medical doctor (AS).

Finally, this study was conducted during a larger research study focussed on refugee youths’ mental wellbeing. Due to this larger project, participating teachers and schools may have been more concerned with refugee students’ mental wellbeing or devoted more time to thinking about it than they would have otherwise. Furthermore, participants were limited to those who volunteered to come to a workshop on supporting refugee students. The participants may therefore be more invested in aiding refugee students than the average teacher. Teachers who didn’t participate may have had less knowledge about or empathy for refugee students and may therefore have been less likely to accommodate refugee students. However, the teachers we talked to told us that the roles they take on to support refugee youths were roles they fill for native students too, so it may be the case that most teachers would have similar beliefs about their role in supporting refugee youths.

## Conclusions and future directions

Through this study, we discovered that teachers think schools and teachers should support the mental health of newcomer students. They are successfully providing this support in many ways. However, we uncovered several issues related to schools’ and teachers’ providing support to refugee youths’ wellbeing. First, schools are not always successfully creating an environment of belonging, perhaps due to language and cultural barriers between students. Second, teachers appear to be providing therapy-like services despite stating this is not their role; this appears to be due to a combination of ambiguity in the boundaries of their role and the fact that the social support systems outside of schools are overwhelmed and do not provide the timely and high-quality support youths need. Finally, teachers feel burdened by the quantity, variability, and emotionally-taxing nature of their work supporting students.

For professionals and researchers concerned with the mental wellbeing of refugee youth, this study provides novel information about the ways schools and teachers believe they are supporting youth and provide encouragement that teachers generally feel committed to supporting refugee youths’ wellbeing. However, it will also be essential to hear firsthand from refugee youths about the challenges they feel are impacting their mental health. It will be important to note the similarities and differences between the perspectives of teachers and youths to identify misunderstandings and missed opportunities for support, and to further explore the bidirectionality between the individual youth and their educational *microsystem*.

Researchers may also wish to further investigate the influence of the parental *microsystem* on refugee youths’ mental wellbeing, raised by our incidental findings on teachers’ views of parents. Further exploring the *mesosystem* level relationship of teachers and parents will allow researchers to identify potential areas of tension where parent and teacher role expectations conflict. Addressing these tensions could improve teacher-parent relationships, which would ultimately lead to improved developmental outcomes for refugee youths.

For interventionists and other mental health practitioners who aim to engage teachers as stakeholders in school-based interventions, our results suggest that schools and teachers *are* generally open to supporting the mental wellbeing of refugee youths. But interventionists should be cautioned that teachers’ stakeholders responsibilities should align with the roles they feel they hold (e.g., roles should be non-therapeutic), and that stakeholder responsibilities should not exacerbate the time-burdens or emotional toll that teachers already experience in supporting refugee youths’ wellbeing.

Finally, these results have implications for practitioners and policy makers. Our work suggests areas where more resources and attention are needed. If language and cultural barriers are indeed contributing to students failing to feel a sense of belonging at school, more resources should be allocated to providing language supports in schools and teaching intercultural competence. Additional resources should also be allocated to social systems outside of schools so that they can provide timely, quality services to youth, thereby easing the burden on teachers. Policies may be developed that better define the boundaries of teachers’ roles when it comes to providing psycho-social support to students. And these policies, along with the allocation of additional resources to schools, would likely ease the burdens of psychosocial support that teachers are experiencing.

## Supplementary Material

Supplemental Material

## Data Availability

The data sets for this study is not open access, but can be made available upon request to the Principal Investigator (FO) and according to the ethical approval.

## References

[cit0001] Bačáková, M., & Closs, A. (2013). Continuing professional development (CPD) as a means to reducing barriers to inclusive education: Research study of the education of refugee children in the Czech Republic. *European Journal of Special Needs Education*, 28(2), 203–15. 10.1080/08856257.2013.778108

[cit0002] Borsch, A. S., Skovdal, M., & Jervelund, S. S. (2019). How a school setting can generate social capital for young refugees: Qualitative insights from a folk high school in Denmark. *Journal of Refugee Studies* 34 1 718–740 . 10.1093/jrs/fez003

[cit0003] Boyson, B., & Short, D. (2012). *Helping newcomer students succeed in secondary schools and beyond*. Centre for Applied Linguistics. http://toolbox2.s3.amazonaws.com/accnt_29013/site_29014/Documents/Tenino_ELLTeacerResources_100913.pdf

[cit0004] Braun, V., & Clarke, V. (2006). Using thematic analysis in psychology. *Qualitative Research in Psychology*, 3(2), 77–101. 10.1191/1478088706qp063oa

[cit0005] Braun, V., & Clarke, V. (2019). Reflecting on reflexive thematic analysis. *Qualitative Research in Sport, Exercise and Health*, 11(4), 589–597. 10.1080/2159676X.2019.1628806

[cit0006] Bronfenbrenner, U. (1977). Toward an experimental ecology of human development. *American Psychologist*, 32(7), 513. 10.1037/0003-066X.32.7.513

[cit0007] Bronstein, I., & Montgomery, P. (2011). Psychological distress in refugee children: A systematic review. *Clinical Child and Family Psychology Review*, 14(1), 44–56. 10.1007/s10567-010-0081-0 21181268

[cit0008] Clarke, V., & Braun, V. (2013). Teaching thematic analysis: Overcoming challenges and developing strategies for effective learning. *The Psychologist*, 26(2 120–123 http://www.thepsychologist.org.uk/archive/archive_home.cfm?volumeID=26&editionID=222&ArticleID=2222).

[cit0009] Correa-Velez, I., Gifford, S. M., & Barnett, A. G. (2010). Longing to belong: Social inclusion and wellbeing among youth with refugee backgrounds in the first three years in Melbourne, Australia. *Social Science & Medicine*, 71(8), 1399–1408. 10.1016/j.socscimed.2010.07.018 20822841

[cit0010] Cristini, F., Scacchi, L., Perkins, D. D., Santinello, M., & Vieno, A. (2011). The influence of discrimination on immigrant adolescents’ depressive symptoms: What buffers its detrimental effects? *Psychosocial Intervention*, 20(3), 243–253. 10.5093/in2011v20n3a2

[cit0011] Denkelaar, M. (2018). *Refugee education in Sweden*. Multi-country Partnership to Enhance the Education of Refugee and Asylum-seeking Youth in Europe. Retrieved August 15, 2021, from https://www.sirius-migrationeducation.org/wp-content/uploads/2018/10/Refugee-Education-in-Sweden-final.pdf

[cit0012] Durbeej, N., McDiarmid, S., Sarkadi, A., Feldman, I., Punamäki, R. L., Kankaanpää, R., Andersen, A., Hilden, P. K., Verelst, A., Derluyn, I., & Osman, F. (2021). Evaluation of a school-based intervention to promote mental health of refugee youth in Sweden (The RefugeesWellSchool Trial): Study protocol for a cluster randomized controlled trial. *Trials*, 22(1), 1–13. 10.1186/s13063-020-04995-8 33509268 PMC7841907

[cit0013] Education Act (Skollag). 2010:800. Retrieved August 15, 2021, from https://www.riksdagen.se/sv/dokument-lagar/dokument/svensk-forfattningssamling/skollag-2010800_sfs-2010-800

[cit0014] Erlandson, P., Strandler, O., & Karlsson, M. R. (2020). A fair game–the neoliberal (re) organisation of social and relational practices in local school settings. *British Journal of Sociology of Education*, 41(3), 410–425. 10.1080/01425692.2019.1707067

[cit0015] Fazel M. (2015). A moment of change: Facilitating refugee children's mental health in UK schools. International Journal of Educational Development, 41 255–261. 10.1016/j.ijedudev.2014.12.006

[cit0016] Fazel, M., Doll, H., & Stein, A. (2009). A school-based mental health intervention for refugee children: An exploratory study. *Clinical Child Psychology and Psychiatry*, 14(2), 297–309. 10.1177/1359104508100128 19293324

[cit0017] Fazel, M., Garcia, J., & Stein, A. (2016). The right location? Experiences of refugee adolescents seen by school-based mental health services. *Clinical Child Psychology and Psychiatry*, 21(3), 368–380. 10.1177/1359104516631606 26907460

[cit0018] Fazel, M., Reed, R. V., Panter-Brick, C., & Stein, A. (2012). Mental health of displaced and refugee children resettled in high-income countries: Risk and protective factors. *The Lancet*, 379(9812), 266–282. 10.1016/S0140-6736(11)60051-2 21835459

[cit0019] Fazel, M., Wheeler, J., & Danesh, J. (2005). Prevalence of serious mental disorder in 7000 refugees resettled in western countries: A systematic review. *The Lancet*, 365(9467), 1309–1314. 10.1016/S0140-6736(05)61027-6 15823380

[cit0020] Fejes, A., & Dahlstedt, M. (2020). Language introduction as a space for the inclusion and exclusion of young asylum seekers in Sweden. *International Journal of Qualitative Studies on Health and Well-being*, 15(sup2), 1761196. 10.1080/17482631.2020.1761196 33297898 PMC7733890

[cit0021] Green, J., & Thorogood, N. (2014). *Qualitative methods for health research* (4th ed.). Sage.

[cit0022] Guo, Y., Maitra, S., & Guo, S. (2019). “I belong to nowhere”: Syrian refugee children’s perspectives on school integration. *Journal of Contemporary Issues in Education*, 14(1), 89–105. 10.20355/jcie29362

[cit0023] Hek, R. (2005). *The experiences and needs of refugee and asylum seeking children in the UK: A literature review* (Birmingham: National Evaluation of the Children’s Fund, University of Birmingham.). https://dera.ioe.ac.uk/5398/1/RR635.pdf

[cit0024] Hoot J L. (2011). Working with very young refugee children in our schools: Implications for the world's teachers. Procedia - Social and Behavioral Sciences, 15 1751–1755. 10.1016/j.sbspro.2011.03.363

[cit0025] Kandemir, H., Karataş, H., Çeri, V., Solmaz, F., Kandemir, S. B., & Solmaz, A. (2018). Prevalence of war-related adverse events, depression and anxiety among Syrian refugee children settled in Turkey. *European Child & Adolescent Psychiatry*, 27(11), 1513–1517. 10.1007/s00787-018-1178-0 29948231

[cit0026] Khamis, V. (2019). Posttraumatic stress disorder and emotion dysregulation among Syrian refugee children and adolescents resettled in Lebanon and Jordan. *Child Abuse & Neglect*, 89, 29–39. 10.1016/j.chiabu.2018.12.013 30612072

[cit0027] Kia-Keating, M., & Ellis, B. H. (2007). Belonging and connection to school in resettlement: Young refugees, school belonging, and psychosocial adjustment. *Clinical Child Psychology and Psychiatry*, 12(1), 29–43. 10.1177/1359104507071052 17375808

[cit0028] Kien, C., Sommer, I., Faustmann, A., Gibson, L., Schneider, M., Krczal, E., Jank, R., Klerings, I., Szelag, M., Kerschner, B., Brattström, P., & Gartlehner, G. (2019). Prevalence of mental disorders in young refugees and asylum seekers in European Countries: A systematic review. *European Child & Adolescent Psychiatry*, 28(10), 1295–1310. 10.1007/s00787-018-1215-z 30151800 PMC6785579

[cit0029] Krueger, R. A. (2014). *Focus groups: A practical guide for applied research*. Sage Publications.

[cit0030] Lindert, J., Von Ehrenstein, O. S., Priebe, S., Mielck, A., & Brähler, E. (2009). Depression and anxiety in labor migrants and refugees: A systematic review and meta-analysis. *Social Science & Medicine*, 69(2), 246–257. 10.1016/j.socscimed.2009.04.032 19539414

[cit0031] Luthar, S. S., Crossman, E. J., & Small, P. J. (2015 Resilience and adversity. Lerner, R. M.). . *Handbook of Child Psychology and Developmental Science: Vol. 3. Socioemotional processes* (New York: Wiley), 247–277. 10.1002/9781118963418.childpsy307

[cit0032] Migrationsverket. (2017). *Asylum decisions, Swedish migration agency, 2016*. Retrieved March 26, 2021, from https://www.migrationsverket.se/download/18.2d998ffc151ac3871592564/1485556054285/Avgjorda%20asyl%C3%A4renden%202016%20-%20Asylum%20decisions%202016.pdf

[cit0033] Ministry of Education & Research. (2016, June). *OECD review of policies to improve the effectiveness of resource use in schools (School resources review). Country background report: Sweden*. Retrieved August 15, 2021, from https://www.oecd.org/education/school/CBR_OECD_SRR_SE-FINAL.pdf

[cit0034] Mock-Muñoz de Luna, C., Granberg, A., Krasnik, A., & Vitus, K. (2020). Towards more equitable education: Meeting health and wellbeing needs of newly arrived migrant and refugee children—perspectives from educators in Denmark and Sweden. *International Journal of Qualitative Studies on Health and Well-being*, 15(sup2), 1773207. 10.1080/17482631.2020.1773207 33297896 PMC7733908

[cit0035] Montgomery, E., & Foldspang, A. (2008). Discrimination, mental problems and social adaptation in young refugees. *European Journal of Public Health*, 18(2), 156–161. 10.1093/eurpub/ckm073 17631490

[cit0036] Moore, S. E., Norman, R. E., Suetani, S., Thomas, H. J., Sly, P. D., & Scott, J. G. (2017). Consequences of bullying victimization in childhood and adolescence: A systematic review and meta-analysis. *World Journal of Psychiatry*, 7(1), 60. 10.5498/wjp.v7.i1.60 28401049 PMC5371173

[cit0037] Morgan, D. L. (2002). Focus group interviewing Gubrium, J. F., and Holstein, J. A. In *Handbook of interview research: Context and method* (Thousand Oaks: Sage) (pp. 141–159).

[cit0038] National Agency for Education. (2021, June). *Support for newly arrived students*. Retrieved August 25, 2021, from https://www.skolverket.se/regler-och-ansvar/ansvar-i-skolfragor/stod-for-nyanlanda-elever

[cit0039] Nowell, L. S., Norris, J. M., White, D. E., & Moules, N. J. (2017). Thematic analysis: Striving to meet the trustworthiness criteria. *International Journal of Qualitative Methods*, 16(1), 160940691773384. 10.1177/1609406917733847

[cit0040] Nyman, M., & Vargar, P. (2020). *Country report: Sweden*. Asylum Information Database. https://asylumineurope.org/wp-content/uploads/2020/05/report-download_aida_se_2019update.pdf

[cit0041] Osman, F., Mohamed, A., Warner, G., & Sarkadi, A. (2020). Longing for a sense of belonging—Somali immigrant adolescents’ experiences of their acculturation efforts in Sweden. *International Journal of Qualitative Studies on Health and Well-being*, 15(sup2), 1784532. 10.1080/17482631.2020.1784532 33297899 PMC7734117

[cit0042] Oxman-Martinez, J., & Choi, Y. R. (2014). Newcomer children: Experiences of inclusion and exclusion, and their outcomes. *Social Inclusion*, 2(4), 23–37. 10.17645/si.v2i4.133

[cit0043] Pastoor, L. D. W. (2017). Reconceptualising refugee education: Exploring the diverse learning contexts of unaccompanied young refugees upon resettlement. *Intercultural Education*, 28(2), 143–164. 10.1080/14675986.2017.1295572

[cit0044] Pastoor, L. (2015). The mediational role of schools in supporting psychosocial transitions among unaccompanied young refugees upon resettlement in Norway. *International Journal of Educational Development*, 41, 245–254. 10.1016/j.ijedudev.2014.10.009

[cit0045] Rendle, K. A., Abramson, C. M., Garrett, S. B., Halley, M. C., & Dohan, D. (2019). Beyond exploratory: A tailored framework for designing and assessing qualitative health research. *BMJ Open*, 9(8), e030123. 10.1136/bmjopen-2019-030123 PMC6720470 31462482

[cit0046] Rizkalla, N., Mallat, N. K., Arafa, R., Adi, S., Soudi, L., & Segal, S. P. (2020). “Children are not children anymore; they are a lost generation”: Adverse physical and mental health consequences on syrian refugee children. *International Journal of Environmental Research and Public Health*, 17(22), 8378. 10.3390/ijerph17228378 PMC7696198 33198333

[cit0047] Rosshandler, Y., Hall, B. J., & Canetti, D. (2016). An application of an ecological framework to understand risk factors of PTSD due to prolonged conflict exposure: Israeli and Palestinian adolescents in the line of fire. *Psychological Trauma: Theory, Research, Practice, and Policy*, 8(5), 641. 10.1037/tra0000124 PMC4982813 26950012

[cit0048] Salari, R., Malekian, C., Linck, L., Kristiansson, R., & Sarkadi, A. (2017). Screening for PTSD symptoms in unaccompanied refugee minors: A test of the CRIES-8 questionnaire in routine care. *Scandinavian Journal of Public Health*, 45(6), 605–611. 10.1177/1403494817715516 28669316

[cit0049] Scherer, N., Hameed, S., Acarturk, C., Deniz, G., Sheikhani, A., Volkan, S., Örücü, A., Pivato, I., Akıncı, İ., Patterson, A., & Polack, S. (2020). Prevalence of common mental disorders among Syrian refugee children and adolescents in Sultanbeyli district, Istanbul: Results of a population-based survey. *Epidemiology and Psychiatric Sciences*, 29, e192. 10.1017/S2045796020001079 33298230 PMC7737189

[cit0050] Schleicher, A. (2018). . In *Valuing our Teachers and Raising their Status*. Paris: OECD Publishing. 10.1787/9789264292697

[cit0051] Şeker, B. D., & Sirkeci, I. (2015). Challenges for refugee children at school in Eastern Turkey. *Economics & Sociology*, 8(4), 122. 10.14254/2071-789X.2015/8-4/9

[cit0052] Sills, A., and Desai, P.(1996, July). Qualitative research amongst ethnic minority communities in Britain. Journal of the Market Research Society 38(3): 247–265.

[cit0053] Steel, Z., Chey, T., Silove, D., Marnane, C., Bryant, R. A., & Van Ommeren, M. (2009). Association of torture and other potentially traumatic events with mental health outcomes among populations exposed to mass conflict and displacement: A systematic review and meta-analysis. *Journal of the American Medical Association*, 302(5), 537–549. 10.1001/jama.2009.1132 19654388

[cit0054] Thapa, A., Cohen, J., Guffey, S., & Higgens-Alessandro, A. (2013). A review of school climate research. *Review of Educational Research*, 83(3), 357–385. 10.3102/0034654313483907

[cit0055] Thijs, J., & Verkuyten, M. (2008). Peer victimization and academic achievement in a multiethnic sample: The role of perceived academic self-efficacy. *Journal of Educational Psychology*, 100(4), 754. 10.1037/a0013155

[cit0056] Thommessen, S. A. O. T., & Todd, B. K. (2018). How do refugee children experience their new situation in England and Denmark? Implications for educational policy and practice. *Children and Youth Services Review*, 85, 228–238. 10.1016/j.childyouth.2017.12.025

[cit0057] Tian, K., & Guang, T. (2017). Exploration of collectivism in contemporary Sweden. *The Journal of Applied Business and Economics*, 19(11/12), 11–27 https://articlegateway.com/index.php/JABE/article/view/774.

[cit0058] Turrini, G., Purgato, M., Ballette, F., Nosè, M., Ostuzzi, G., & Barbui, C. (2017). Common mental disorders in asylum seekers and refugees: Umbrella review of prevalence and intervention studies. *International Journal of Mental Health Systems*, 11(51 1–14). 10.1186/s13033-017-0156-0 28855963 PMC5571637

[cit0059] Tyrer, R. A., Fazel, M., & Chao, L. (2014). School and community-based interventions for refugee and asylum seeking children: A systematic review. *PloS ONE*, 9(2), e89359. 10.1371/journal.pone.0089359 24586715 PMC3933416

[cit0060] UNICEF. (2016, September). *Uprooted: The growing crisis for refugee and migrant children*. https://www.unicef.org/publications/files/Uprooted_growing_crisis_for_refugee_and_migrant_children.pdf

[cit0061] United Nations High Commissioner for Refugees (UNHCR). (2020, June 18). *Global trends: Forced displacement in 2019*. https://www.unhcr.org/5ee200e37.pdf

[cit0062] Wong, J. P.-H., & Poon, M. K.-L. (2010). Bringing translation out of the shadows: Translation as an issue of methodological significance in cross-cultural qualitative research. *Journal of Transcultural Nursing*, 21(2), 151–158. 10.1177/1043659609357637 20220035

