# Supplementary material for: Schools’ and teachers’ roles and challenges in supporting the mental wellbeing of refugee youths: a qualitative study with Swedish teachers
Source: Int J Qual Stud Health Well-being. 2021 Dec 1;17(1):2007568. doi: 10.1080/17482631.2021.2007568 (PMC8648014; doi:10.1080/17482631.2021.2007568)
Supplement: Supplemental Material [file ZQHW_A_2007568_SM1147.docx]

## Supplementary Information

***Focus Group Questions***

| Topic | Questions |
| --- | --- |
| Wellbeing within Schools | How would you describe the wellbeing of refugee/migrant students in your classroom?  How do you perceive that their situation affects their mental health and wellbeing? (limitations, challenges, resilience)  How would you describe the school's role in the refugee students’ health? What is your role in school in children's health?  How do you feel you can support students with refugee experience?  What are you lacking in order to be able to keep up with their resilience? What support would you need in that case?  Which factors could contribute to a positive school environment?  Are there other reflections that you want to bring up? |
| Wellbeing within a Broader Social Context | What expectations do you have for your refugee students’ futures? Are they differing [from other students]?  How do you feel their specific situation impacts their wellbeing?  Are there any other reflections you want to share? |
| Larger Training | What are your expectations for this course that you will be taking soon?  As educators, how do you think this course could help refugee students? |
